# Journalistic Notes

**Published:** 1890-11

**Authors:** 


### Journalistic Notes.

The October number of *The Old Homestead*, the southern literary, fashion, and domestic monthly, published at Savannah, Ga., is up to its usual standing. It has complete and serial stories, poems and essays by some of the most brilliant southern writers, while its fashion department, handsomely illustrated, is one of the best in the country. *The Old Homestead* is the literary magazine of the south, and its freedom from erratic and sensational matter makes it a welcome visitor in the most refined homes. Its only purpose is to encourage literature and to elevate and refine. It is a magazine of forty pages 11x15, with a subscription price of $1 per year ; sample copy free. Davis Bros., publishers, Savannah.

The University Medical Magazine.—Is it too scientific ?— The success of the *University Medical Magazine* was from the beginning a foregone conclusion. With a list of contributors heading the profession of this country, the popularity of the *Magazine* has steadily extended until the number of subscribers from the medical school, in whose interest it was originally started, is far exceeded by those of other schools from all sections of the country. Since the issue of the first number the harshest criticism upon the *Magazine* has been that it is too scientific. If by this is meant that its teachings are carefully weighed and are accurate, and that no statements of fact are made without the support of weighty authority for their truth, then this criticism is correct. If, on the other hand, it is implied that the *Magazine* is devoted to abstract theory and investigation, which is utterly devoid of practical application, then the *Magazine* is not too scientific.

---

THE MONIST.— *A New Quarterly Magazine of Philosophy, Science, Religion and Sociology.*— On October the first, The Open Court Publishing Co., of Chicago, began the publication of a new magazine with the above title.

The first number contained articles by Prof. E. D. Cope, of Philadelphia; Prof. George J. Romanes, of London; M. Alfred Binet, of Paris; Prof. Ernst Mach, of Prague; Max Dessoir, of Berlin, and Dr. Paul Carus, of Chicago.

The foreign correspondence and the departments for the general review of foreign philosophical and scientific literature will be conducted, for Italy, by Prof. C. Lombroso, the criminologist; for France, by Lucien Arréat, the critic of the *Revue Philosophique;* for the northern countries, by Prof. Harald Höffding, of Copenhagen; for Germany, by Prof. F. Jodl, of Prague, and others. Reviews of American and English books will appear separately.

Articles will appear in *The Monist* by Prof. Joseph Le Conte, Prof. William James, Charles S. Pierce, Prof. Max Müller, Prof. Ernst Hæckel, and Th. Ribot.

The magazine will be devoted to the establishment and illustration of the principles of Monism in Philosophy, Exact Science, Religion and Sociology. So far as the fulfillment of this aim will allow, it will bear a popular character; publishing articles of general interest, as well as those of a more special character. The Open Court Publishing Co., 169-175 La Salle street, Chicago.

---

THE STORY OF A MAGAZINE.— A most interesting story of the conception and growth of *The Ladies' Home Journal*, of Philadelphia, with portraits and sketches of its proprietor and editor, has been prepared by that magazine, in pamphlet form, and will be sent free to any who will write for a copy.

